# Pivotal functions and impact of long con-coding RNAs on cellular processes and genome integrity

**DOI:** 10.1186/s12929-024-01038-1

**Published:** 2024-05-14

**Authors:** Siddhant Sharma, Aicha Asma Houfani, Leonard J. Foster

**Affiliations:** 1 Department of Chemical and Biological Engineering, University of British Columbia, Vancouver, BC V6T 1Z3 Canada; 2 Michael Smith Laboratories and Department of Biochemistry and Molecular Biology, University of British Columbia, 2185 E Mall, Vancouver, BC V6T 1Z4 Canada

**Keywords:** Long non-coding RNAs, Cell division, Cell cycle, Immune responses, Hematopoiesis

## Abstract

Recent advances in uncovering the mysteries of the human genome suggest that long non-coding RNAs (lncRNAs) are important regulatory components. Although lncRNAs are known to affect gene transcription, their mechanisms and biological implications are still unclear. Experimental research has shown that lncRNA synthesis, subcellular localization, and interactions with macromolecules like DNA, other RNAs, or proteins can all have an impact on gene expression in various biological processes. In this review, we highlight and discuss the major mechanisms through which lncRNAs function as master regulators of the human genome. Specifically, the objective of our review is to examine how lncRNAs regulate different processes like cell division, cell cycle, and immune responses, and unravel their roles in maintaining genomic architecture and integrity.

## Introduction

**Fig. 1:**
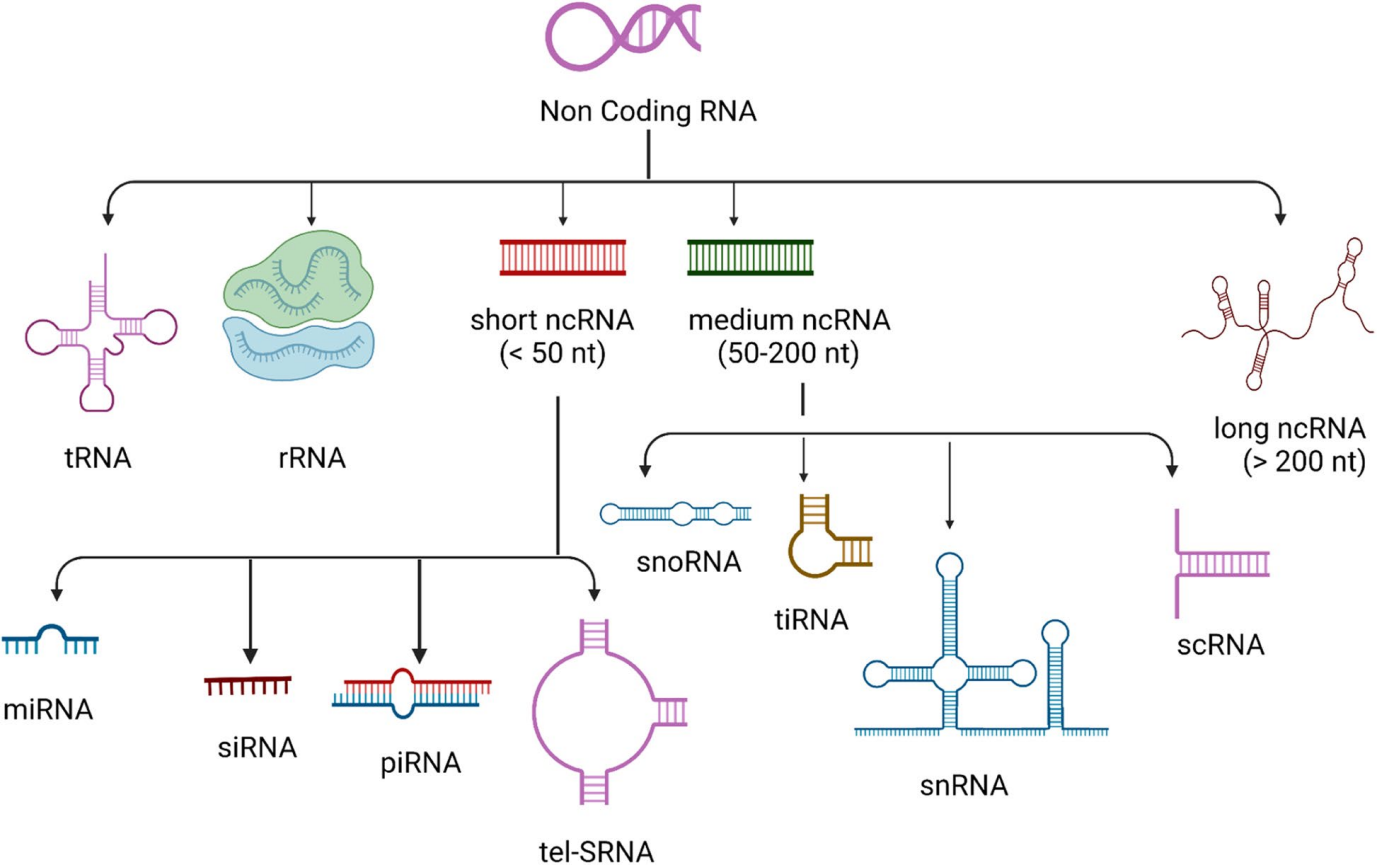
Classification of ncRNAs. The above figure summarizes the different species of non-coding RNAs present in the human genome based on function and length of nucleotide base. ncRNAs are divided into multiple species: Transfer RNAs (tRNAs), ribosomal RNAs (rRNAs), short (20–50 nucleotides), medium (50–200 nucleotides), and long (> 200 nucleotides). The short ncRNA group is further subdivided into microRNAs (miRNAs), small interfering RNAs (siRNAs), piwi-interacting RNAs (piRNAs), and telomere-specific RNAs (tel-sRNAs). The medium ncRNA group contains 4 species: small nucleolar RNAs (snoRNAs), tRNA-derived stress-induced RNAs (tiRNAs), small nuclear RNAs (snRNAs), and small cytoplasmic RNAs (scRNAs) [19, 46, 83]. lncRNAs are ncRNAs that are longer than 200 nucleotides and lack appreciable protein translation [3, 21, 50]

The Human Genome Project discovered that only ~ 2% of the genome is transcribed and translated into proteins. The bulk of RNA molecules exist as non-coding RNAs (ncRNAs) that do not convert into proteins. ncRNAs are classified based on their structure and regulatory role in biological processes. They are divided into multiple species (Fig. 1): transfer RNAs (tRNAs), ribosomal RNAs (rRNAs), short (20–50 nucleotides), medium (50–200 nucleotides), and long (> 200 nucleotides). The short ncRNA group can be further subdivided into microRNAs (miRNAs), small interfering RNAs (siRNAs), piwi-interacting RNAs (piRNAs), and telomere-specific RNAs (tel-sRNAs). The medium ncRNA group contains 4 species: small nucleolar RNAs (snoRNAs), tRNA-derived stress-induced RNAs (tiRNAs), small nuclear RNAs (snRNAs), and small cytoplasmic RNAs (scRNAs) [19, 46, 83].

Long non-coding RNAs **(**lncRNAs) are ncRNAs that are longer than 200 nucleotides and lack notable protein translation [3, 21, 50]. Like mRNAs, most lncRNAs are produced by RNA Polymerase-II (Pol-II) transcription, a complex process that includes but is not limited to, splicing, cleavage/polyadenylation (C/P) export, and surveillance. lncRNAs frequently consist of multiple exons, typically have 7-methyl guanosine (m^7^G) at their 5' ends, and are polyadenylated at their 3' ends [67, 102]. Interestingly, lncRNA-encoding regions of the genome share many similar properties with their translated counterpart mRNAs. For example, the abundance of repressive histone modifications at promoter regions, regulating the chromatin structure of a target locus [37], and the presence of epigenetic markers along transcribed regions [62, 107]. Growing advances in proteomic technologies and transcriptomic analysis have revealed the important role that lncRNAs play in regulating diverse cellular processes, including the cell cycle [44, 115, 134], cell division [51, 124], DNA repair [97, 138], post-translational modifications (PTMs) [33, 59], stem cell pluripotency [13, 132], and RNA splicing [42, 131].

Our goal in this review is to integrate and synthesize what is known about the role of lncRNAs in regulating various cellular processes including immune responses, cell division, cell cycle, genome architecture, and hematopoietic differentiation. It's interesting to note that, despite their apparent differences, these systems are all vital to ensure healthy biological and metabolic functioning. Consequently, comprehending the regulatory functions of lncRNAs in modulating these cellular activities is essential to accelerate the creation of innovative therapeutic strategies for treating a variety of illnesses**.** Herein, we provide a concise overview of the current understanding of various biological characteristics of lncRNAs, followed by a detailed review of their contributions to mediating the aforementioned biological processes. By understanding the roles of distinct lncRNAs, we will be able to acquire a deeper insight into the connections between different fundamental systems governing the human genome.

## Biological characteristics of lncRNAs

### Classification of lncRNAs

Studies based on next-generation RNA sequencing (RNA-seq) have divided lncRNAs into two categories according to where they are located in the human genome relative to the nearest protein-coding gene (PCG): intergenic or intronic. The DNA sequence in between genes is known as an intergenic region while intronic regions are transcripts found inside genes themselves. Intergenic lncRNAs are found in the intergenic regions between 2 PCGs while intron lncRNAs are formed solely due to the transcription of introns of PCGs [66]. Based on their orientation with respect to PCG strands, lncRNAs are classified as sense (transcribed as a sense RNA strand of a PCG) or antisense (transcribed as an antisense RNA strand of a PCG) [74]. lncRNAs transcribed at a distance of < 1000 bases away on the opposite strand from the promoter region of a PCG are called bidirectional lncRNAs [4, 50]. Further classification splits lncRNAs based on the DNA strand's product orientation [4, 50]. To summarize, the entire lncRNA network in the human genome consists of a majority of antisense lncRNAs followed closely by intronic, bidirectional, and overlapping transcriptions [70].

### Structure–function relationship of lncRNAs

A prominent feature of lncRNAs is their capability of assembling into thermodynamically stable and higher-order structures [70], and understanding the structure of various lncRNA transcripts has helped to delineate their function. For instance, the genetic stability of the lncRNA XIST is ensured by specific functional elements on its 3’ end. XIST contains six distinct, conserved structural motifs called A-F tandem repeat regions. These regions function as organized RNA motifs on XIST that facilitate its stable interactions with different proteins [86, 99]. In addition, lncRNAs experience extensive alternative splicing [142] and, consequently, exist in many isoforms. Illustratively, the lncRNA MEG3 is a tumor suppressor transcript located on the 14q32.3 chromosome in humans and is frequently underexpressed in an array of malignancies [128]. There exist at least twelve distinct isoforms of the lncRNA MEG3 and the expression of any isoform triggers p53 transcription, leading to tumor suppression. Computational modeling and deletion studies revealed that three different secondary structural motifs (M1-3) are present on each MEG3 isoform and M2-M3 are particularly crucial for p53 gene transactivation, further confirming that an organized, and stable lncRNA structure is necessary for mediating healthy biological functions [136].

### Subcellular localization of lncRNAs

lncRNAs interact with many signaling receptors [129, 140] and proteins [85, 135], and therefore a greater understanding of the subcellular localization of lncRNAs is important to comprehend and generalize their various action mechanisms for regulating biological processes [8, 11]. The current understanding of the subcellular localization of lncRNAs is limited and remains to be fully elucidated. lncRNAs' subcellular localization is influenced by several factors, including structural motifs and sequences that direct the process of protein binding [34]. Most lncRNAs are not expressed in appreciably detectable quantities, which has created challenges in organizing their structure, and exploring their roles [127]. Nevertheless, certain important characteristics have been identified that are common to a majority of lncRNAs. For instance, over 81% of lncRNAs are poorly conserved and contain fewer exons relative to mRNAs [24, 102] due to less efficient and chaotic splicing relative to mRNAs [103].

A significant portion of lncRNAs are preferentially localized in the cell nucleus (known as nuclear lncRNAs). Nuclear lncRNAs are associated with many molecular processes, including chromatin remodeling, transcriptional and post-transcriptional modifications, and functioning as biomolecular condensates [67, 101]. Despite a dominant preferential nuclear localization, many lncRNAs do function outside of the nucleus, in the cytoplasm, and with ribosomes. For instance, a study by (van [116], on the human cell line LS-174 T-pTER-*β*-catenin revealed that a significant portion of lncRNAs were associated with ribosomes (lncRNA H19 and TUG1) and the cytosol, which indicated that many unexplored functions of lncRNAs exist which can be a significant topic for future research. For example, RNA molecules that catalyze biochemical reactions are referred to as ribozymes, and a relatively new area of research focuses on the role of lncRNAs as ribozymes in regulating gene expression. Hovlinc is an intriguing ribozyme that was found recently and is embedded in the 96 kb lncRNA ID210. Even though Hovlinc's catalytic qualities are yet unknown, the novel finding by [16] provides a wealth of opportunities for further investigation into and the function of lncRNAs as RNA catalytic domains. Furthermore, since ID210 is currently the only known lncRNA with a ribozyme structure, it will be interesting to see if other lncRNAs have ribozymes and investigate what unique capabilities they possess.

Mechanistically, the NXF1 pathway is responsible for the nuclear export of long-A/U-rich RNA transcripts with a low exon count to the cytoplasm [141]. Since there are fewer exons in lncRNAs in comparison to their mRNA counterparts, lncRNAs may be able to employ this pathway to exit the nucleus and consequently get assigned to various organelles, such as the mitochondria and RNA-binding proteins (RBPs) [102].

Indeed, lncRNAs are also known to be localized in the mitochondria, and their expression patterns are controlled by nuclear proteins [30, 89]. This assertion is supported by the discovery of three distinct and stable lncRNAs (lncND5, lncND6, and lncCyt b RNA) in the mitochondrial genome of HeLa cells. Furthermore, the protein MRPP1 is essential for regulating the mitochondrial genome due to its key role in mitochondrial respiration [94]. The altercation of MRPP1 protein is strongly associated with a significant reduction in the levels of all 3 lncRNAs and it is believed that these specific lncRNAs may play a role in stabilizing ND5/6, and Cyt b expressions due to their ability to form intermolecular duplexes [89].

## Mechanisms of action of lncRNAs for gene regulation

### lncRNAs as chromatin organizers and molecular scaffolds

lncRNAs interact with histone-modifying complexes and DNA methylation agents in cis (local to the site of lncRNA production) or trans (genes at distant target sites) and guide them to target genomic loci, allowing them to execute their biological functions. Primarily, lncRNAs covalently regulate chromatin organization by partnering with chromatin-modifying enzymes causing changes in chromatin organization [17].

lncRNAs function as molecular scaffolds to provide a platform for facilitating interactions amongst various molecules and proteins to group diverse enzymes, RBPs, and other regulatory molecular factors. RBPs subsequently target specific genomic regions and alter or regulate gene expression [4]. The lncRNA scaffolding effect also assists in the aggregation of various macromolecular structures like the PRC complex, histone methyltransferases, demethylases, and even transcription factors which supports the incorporation of information in multiple signaling pathways [29].

### Direct interactions between lncRNAs and miRNAs

lncRNAs execute gene transcription by presenting “decoy” binding sites to sequester various transcription factors and protein-chromatin modifying complexes. Mechanistically, upon activation, lncRNAs will bind to and displace their protein targets [121] without transcriptional interference [24]. This lncRNA-mediated sequestration is achieved by either competing with other RNA complexes or forcing RBPs into a dormant state. Kinetically, the efficiency of gene regulation by decoy action of lncRNAs is strongly dependent on the concentration of lncRNAs and the participating RBPs, and the affinity, and stability of the subsequent interaction amongst them [108].

lncRNAs act as competitive endogenous RNAs (ceRNAs), which is another way they can act as decoys. ceRNAs can significantly constrain the ability of miRNAs to communicate with their target mRNAs by either sequestering miRNA elements or barricading miRNA binding sites altogether leading to alterations in downstream axial protein mRNA expressions [123].

### lncRNAs as signaling molecules

lncRNAs mediate signal transduction among cells by interacting with a variety of receptors, protein kinases, and transcription factors. Cross communication amongst signaling pathways and signaling cascades are controlled by lncRNAs in 6 ways. For further understanding of these specific 6 pathways, we recommend that the readers examine an excellent review presented by [58].

## lncRNAs mediate innate and trained immune responses

### Inflammatory response and antiviral defense by lncRNAs

**Fig. 2:**
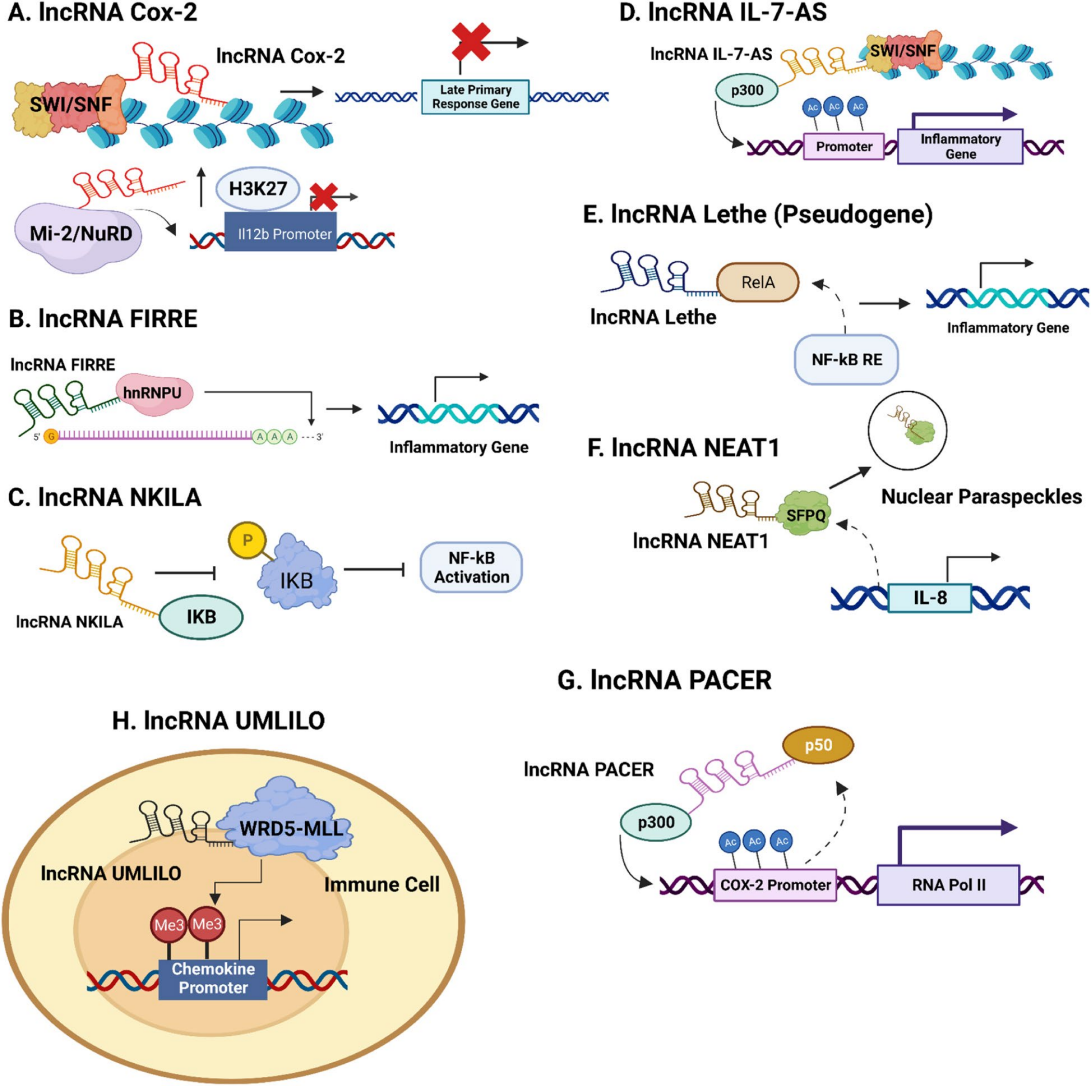
Immune Response and Trained Immunity Mediation By lncRNAs. **A** | lncRNA Cox-2 modulates the transcription of late primary genes by SWI/SNF-associated chromatin remodeling [45]. By recruiting the Mi-2/NuRD suppressor complex to the Il12b promoter in the epithelium, increased H3K27 demethylation is observed and consequently the Il12b gene becomes trans-hindered [114]. **B** | In response to LPS stimulation, lncRNA FIRRE interacts with hnRNPU to control the stability of mRNAs belonging to specific inflammatory genes by exerting its effects on the AU-rich regions of those mRNAs [65]. **C** | lncRNA NKILA negatively controls the NF-kB signaling pathway by directly binding at the p65- N terminal region of IkB to suppress IKK directed IκB phosphorylation and this action is critical to suppress breast cancer metastasis [20]. **D** | lncRNA, IL-7-AS forms a complex with the histone acetyltransferase p300 to increase histone acetylation at the promoting regions of the genetic loci of CCL2, CCL5, and CCL7 genes. It also functions as a chromatin remodeler to regulate the assembly of the SWI/SNF complex to the promoter regions of the target inflammatory genes [61]. **E** | lncRNA lethe hinders the communication between RelA and NF-kB response elements to negatively regulate NF-kB signaling [90]. **F** | The lncRNA NEAT1 selectively displaces the SFPQ protein to nuclear paraspeckles for reactivating IL8 transcription for mediating immune response [49]. **G** | lncRNA PACER increases acetylation at the COX2 promoter region by physically dislocating the p50 repressive complex from the promoting region of COX2 and recruiting the histone acetyltransferase p300 [54]. **H** | lncRNA UMLILO guides the WRD5-MLL complex and cis-directs it to CXCL chemokine promoters for conferring trained immunity in mouse chemokine models by activating their H3K4me3 genomic priming [25]

An inflammatory response is the first line of defense in combating invasive pathogens. An inflammatory invigoration elicits the transcription of multiple inflammatory genes that subsequently execute assigned functions like antimicrobial activity [1]. Inflammatory genes are classified as primary response genes and secondary response genes. While most secondary response genes and some primary response genes require SWI/SNF complex remodeling for induction, the bulk of primary response genes have CpG island promoters and are triggered by TLR ligands and TNF [5, 28]. lncRNAs are known to attune the expressions of various genes to regulate inflammatory response (Fig. 2) (Table 1). A well-known example is the lncRNA COX-2, highly expressed in murine macrophages, which governs SWI/SNF-associated chromatin remodeling to control the transcription of late primary response genes [45]. Additionally, in the epithelium, the recruitment of the Mi-2/NuRD suppressor complex to the Il12b promoter is facilitated by COX-2. Consequently, H3K27 demethylation is heightened and hence the Il12b gene becomes trans-hindered [114].

**Table 1:** Role of lncRNAs in innate and trained immune responses

Long Non-Coding RNA	Target Proteins and Genes	Mechanism of Action	References
COX-2	SWI/SNF, Mi-2/NuRD	Regulates transcription of late primary response genes by SWI/SNF chromatin remodeling Facilitates recruitment of Mi-2/NuRD complex to Il12b promoter for trans-hindering the latter’s expression in the epithelium	[45, 114]
FIRRE	hnRNPU	Controls the stability of various mRNAs belonging to specific inflammatory genes found in macrophages/ intestinal epithelial cells	[65]
NKILA	p65 terminal of IkB	Antagonizes NF-kB signaling by binding to the p65 terminal of IkB for suppressing IkB phosphorylation	[20]
IL-7-AS	p300, SWI/SNF	Interacts with p300 to enhance histone acetylation on CCL2, CCL5, and CCL7 loci. Modulates SWI/SNF chromatin remodeling at the promoter regions of the aforementioned genes	[61]
Lethe (Pseudogene)	RelA	Blocks the interaction of RelA with NF-kB response elements to negatively regulate NF-kB signaling pathway for mediating inflammatory response	[90]
NEAT1	SFPQ protein	Rescues IL-8 transcription by relocalizing SFPQ to nuclear paraspeckles	[49]
UMLILO	WRD5-MLL	Guides the WRD5-MLL complex to CXCL promoters to activate H3K4me3 genomic priming	[25]

For the host to mount an effective response against viral invasion, transcription factors that are triggered by the innate immune system are indispensable. For example, the transcription factor IRF3 activates and upregulates IFNβ production to limit viral replication in the host and quickly triggers death in viral cells [32]. The AP-1 transcription factor JunB is important for the classical and alternative activation of macrophages for heightened immune responses against microbial attacks [27]. In addition, the activation of both transcription factors JunB and c-Jun is necessary for increased dendritic cell development in the body for a heightened immune response [27, 79]. Finally, the p65 (RelA) transcription factor is a critical regulator of innate immunity as it forms a heterodimer with p50 to stimulate the production of various inflammatory cytokines to regulate immune response [31].

Interestingly, lncRNAs are known to interact and control the expressions of many such transcription factors transcribed by the immune system to regulate immune responses. Various lncRNAs located proximally close to immune response PCGs are overexpressed in response to LPS stimulation and their role in mediating innate immune response is more structural than functional. It is quite interesting to note that the structure of LPS-induced lncRNAs is such that they have at least one site that favors binding with only one of the four specific transcription factors of p65, JunB, c-Jun, and IRF3 [137] and this actively plays a critical role in regulating the expression of immune system associated transcription factors and therefore by extension innate immunity. [65] revealed that the lncRNA FIRRE utilized PTMs to regulate expressions of VCAM1 and IL12p40 inflammatory genes. Following lipopolysaccharide (LPS) stimulation, FIRRE interacts with heterogeneous nuclear ribonucleoprotein U (hnRNPU) to co-regulate and enhance the stability of VCAM1 and IL12p40 mRNAs by facilitating the binding of hnRNPU to VCAM 1 and IL12p40 mRNA AU-rich regions.

A study by [59], highlighted that the lncRNA NKILA suppresses breast cancer metastasis by acting as an NF-kB inhibitor. Dysregulations of NF-kB signaling are a critical component in the development of various cancers [20], and NKILA suppresses the NF-kB signaling pathway by obstructing IKK directed IκB phosphorylation through direct binding at the p65—N terminal region of IkB. Curiously, another lncRNA, IL-7-AS, stimulates the transcription of various inflammatory genes like CCL2, CCL5, and CCL7 through a dual mechanism. Firstly, IL-7-AS forms a complex with the histone acetyltransferase p300 to increase histone acetylation at the promoters of these inflammatory genes. Secondly, it acts as a chromatin remodeler to control the assembly of the SWI/SNF complex at its target inflammatory genes' promoter regions [61].

The role of pseudogenes in gene transcription is highly underrated, and they are often ignored when taking into account what factors drive transcriptional regulation. Pseudogenes are transcripts that harbor defective gene copies and are believed to possess little to no functional relevance to the functioning of the human genome, however, with greater advances in biomedical technologies, this notion seems to be challenged [12]. More so what is fascinating is that certain lncRNAs exist as pseudogenes for regulating immune responses. An excellent study by [90] highlighted the role of the lncRNA lethe in mediating inflammatory response. Lethe binds to RelA-RelA homodimers, inhibiting RelA's DNA binding to multiple NF-kB signaling sites and thereby adversely affecting NF-kB signaling.

### Protein complex(es) relocalization by lncRNAs

lncRNAs can sequester proteins from their native habitat and control their subcellular localization to regulate innate immune processes. For instance, the lncRNA NEAT1 reactivates IL8 transcription by selectively relocating the SFPQ protein, an active repressor of IL8, to nuclear paraspeckles from the IL8 promoter region. In detail, on exposure to poly I:C treatment, SFPQ relocalizes from the promoter region of IL8 to NEAT1 effectively boosting the production of nuclear paraspeckles that cause transactivation of IL8 mRNA expressions. The poly I:C induced IL8 mRNA activation is rendered null and void on NEAT1 silencing [49].

PACER is an antisense lncRNA located upstream of the COX-2 transcription start site (TSS) [75]. Interestingly, CTCF/cohesin is responsible for inducing the expression of the lncRNA PACER. Mechanistically, PACER interacts with the p50 repressor complex and dislocates it from the promoter of COX-2, and recruits the histone acetyltransferase p300 to increase acetylation at the COX-2 promoter. This histone modification catalyzes the assembly and transcription of RNA Polymerase II (RNA Pol II) structures. This commendable discovery by [54] provides various novel insights into the functioning of lncRNAs. First of all, it reveals that factors regulating genomic architecture can also govern the production of lncRNAs, which in turn affects chromatin organization (discussed ahead in Sect. 5. of our review). Second, lncRNAs can sequester protein repressor complexes from target promoter regions in addition to competing with miRNA elements as ceRNAs.

### Trained immunity regulation by lncRNAs

Trained immunity is a form of immunological memory that is generated in immune cells that are often continuously exposed to microbial attacks, consequently enhancing their transcriptional response to future pathogen invasions [26, 77]. Intriguingly, the lncRNA UMLILO acts as a guide molecule for the WRD5-MLL complex and cis-directs it to CXCL chemokine promoters for activating their H3K4me3 genomic priming inducing immunity training in CXCL genes present in the mouse chemokine TAD [25]. What continues to remain elusive, though, is that apart from UMLILO, very little is known about how lncRNAs are associated with trained immunity and a better understanding would open up new frontiers of knowledge about the functional mechanisms of lncRNAs.

## 3D Genome architecture modulation by lncRNAs

### lncRNA-CTCF Interactions regulate 3D genome structure

**Fig. 3:**
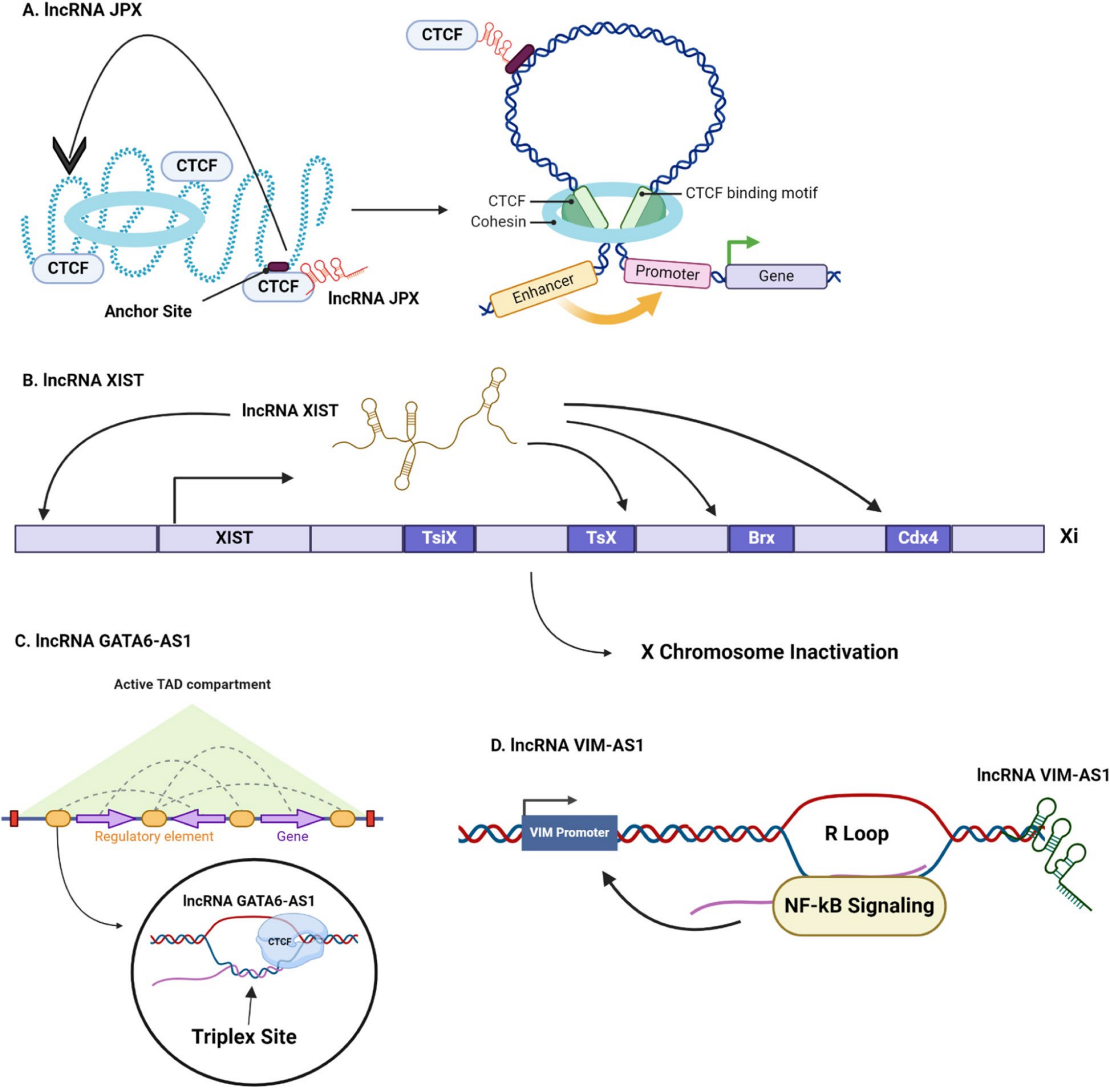
Role of lncRNAs in Regulating Genome Architecture. **A** | lncRNA JPX controls CTCF-DNA communications at low-affinity CTCF binding sites related to genome development [80]. **B** | lncRNA XIST employs a proximal search approach to execute X chromosome inactivation by first identifying favorable binding sites on the X chromosome and then relocalizing the attacked sites closer to its growing locus [23]. **C** | lncRNA GATA6-AS1 has preferential non-randomly aligned triplex sites at the boundaries of topologically associated chromatin domains (TADs) and potentially also interacts with CTCF [100]. **D** | lncRNA transcript VIM-AS1 modulates transcriptional activity by designing an R loop in the VIM promoter region and through the NF-kB signaling pathway [7]

The architecture of the human genome is a critical regulator of its dynamic nature, which further facilitates cellular development and functions. CCCTC-binding factor (CTCF), is the master engineer of genome organization and chromatin remodeling. As a transcription factor, CTCF mediates genome organization by serving as a scaffold on which different molecules, such as RNA Pol II, other transcription factors, and cohesion, assemble for the formation of chromatin loops, which further facilitate gene activation and repression [40]. The human genome is a hierarchically-designed, 3D structure, containing chromatin-interacting domains, nuclear compartments, and short-ranged cis-interactions [93]. While the interactions between CTCF and many transcription factors and cohesions shape genome design and regulate its functions, accumulating evidence suggests that lncRNAs also have a crucial role in organizing the structural motifs of the human genome (Fig. 3).

Studies reveal that lnRNA-CTCF interactions lead to the formation of chromatin loops. The lncRNA JPX is primarily responsible for epigenetically activating and regulating the expression of the lncRNA XIST to facilitate X chromosome inactivation [111]. Interestingly, the traditional notion that the lncRNA JPX is exclusively associated with X inactivation was revised by [80]. JPX dominates over CTCF-DNA communications at low-affinity CTCF binding sites related to genome development. Therefore, JPX can selectively choose appropriate anchor sites for CTCF, and the knockdown of JPX causes deracination of approximately 72–75% of chromosome loops and mutations in more than 700 genes.

Topologically associated chromatin domains (TADs) are the foundational blocks of the 3D human genome, and domains that directly border TADs play an important role in governing gene expression by obstructing the recruitment of cis-regulatory transcripts to their target genes. Disruption of TAD boundaries is the basis for a variety of rare genetic disorders [68]. Recently, [100] showed that the lncRNA GATA6-AS1 had non-randomly aligned triplex sites preferentially at the boundaries of topologically associated chromatin domains (TADs). It was discovered in the study that CTCF played a significant role in TAD specification, and possessed the capability of interacting with GATA6-AS1.

### XIST mediated X chromosome inactivation

The lncRNA XIST plays an important role in X chromosome inactivation for regulating gene expression in female mammals. Interestingly, [23], explained that XIST coats the entire X chromosome by first identifying initial favorable binding sites. By taking advantage of the 3D structure of the X chromosome, XIST relocalizes the attacked sites closer to its growing locus and gradually encompasses the entire structure of the X chromosome consequently silencing its activation. It is anticipated that additional lncRNAs may employ a comparable method to affix subcellular nuclear domains to their target sites to modify the chromatin structure and control transcription.

Deletion of XIST is associated with a domino effect culminating in carcinogenesis due to X reactivation which causes genome-wide dysregulation resulting in aberrant hematopoiesis at extramedullary sites resulting in myelodysplastic and myeloproliferative neoplasms (MDP/MDN), further resulting in leukemogenesis [133].

### Transcriptional regulation by lncRNAs as enhancers

Enhancers recruit RNA Pol II systems to activate the transcription of lncRNAs, confusingly referred to as eRNAs or elncRNAs which are now appreciated for harboring myriad abilities for regulating gene expression [52]. As evidenced by notable eRNA expressions at chromatin loop anchoring sites, recent research by [41] suggested that eRNAs are closely linked to controlling the design of chromatin loops. YY1 is a transcription factor that forms dimer complexes by directly binding to enhancer-promoter elements to facilitate structural interactions between both DNA elements [68, 125]. eRNAs recruit YY1 to their respective, unique chromatin expressions present on their enhancer regions that further promote the communication between the enhancer regions associated with eRNAs and their target promoters. Due to the effective functioning of only a few eRNAs, further experimental studies are needed to generalize whether other eRNAs also recruit YY1 to facilitate the formation of chromatin loops.

### Role of lncRNAs in R loop formation

Experimental studies have revealed that lncRNAs are also actively involved in the formation of RNA–DNA triple helices known as R loops [73]. R loops have conventionally been viewed as harmful and thought to endanger genome integrity due to their capability of inducing DNA damage when not properly folded into a stable structure. Recent findings, however, have challenged this paradigm and reconsidered these traditional viewpoints as they show that these structures can function as essential genetic modulators. For example, The antisense lncRNA transcript VIM-AS1 designs an R loop in the VIM promoter region and activates transcriptional activity via the NF-kB signaling pathway [7].

### lncRNA expressions in cell division and cytoskeleton architecture

**Fig. 4:**
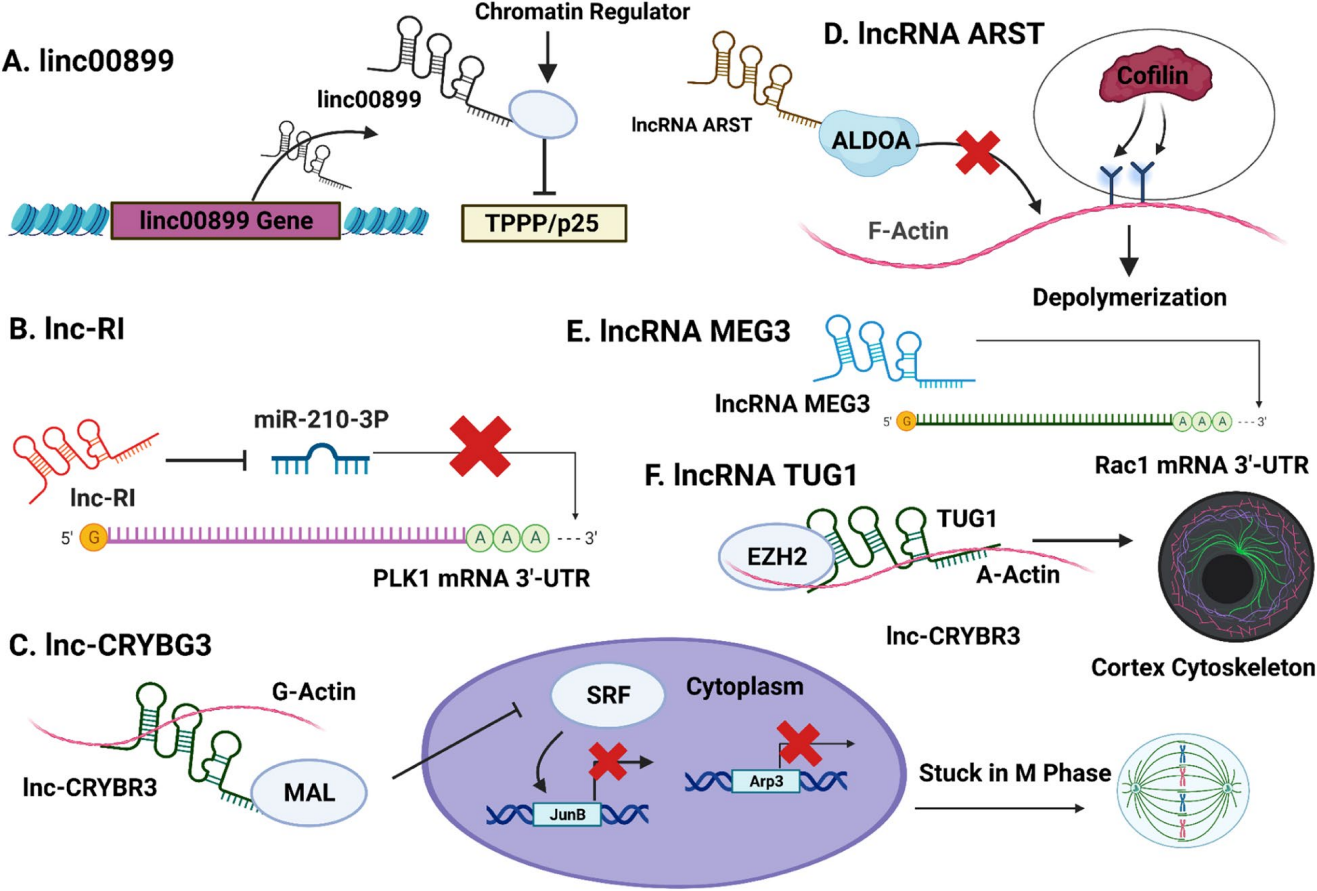
lncRNAs Regulate Mitosis and Cytoskeleton Architecture During Cell. Division. **A** | lncRNA linc00899 suppresses TPPP levels for fine-tuning TPPP/p25 expression to regulate mitosis. Linc00899 possibly physically interacts with various chromatin regulators and since its transcription site is spatially close to TPPP in the cell nucleus, it can act quickly and reduce TPPP levels [104]. **B** | lncRNA—RI sequesters miRNA-210-3p to protect the 3’ UTR mRNA of the PLK1 gene to control cell division [124]. **C** | LNC CRYBG3 prevents the accumulation of MAL in the cell nucleus by interacting with and binding to G-actin and MAL,thereby inactivating SRF and its downstream genes. Consequently, the cells are unable to form a contractile network (CN) and are permanently stuck in the M phase halting further cell division [84]. **D** | lncRNA ARST disturbs the communication between actin fibers and ALDOA which exposes F-actin binding sites to cofilin which causes the cytoskeleton’s depolymerization and consequently the development of new actin fiber elements to get severely hindered [106]. **E** | lncRNA MEG3 negatively regulates the expression of the Rac1 gene by interacting with its 3’-UTR mRNA which results in the inhibition of thyroid carcinoma metastasis [120]. **F** | lncRNA TUG1 facilitates cortex cytoskeleton formation by binding to both α-actin and EZH2 to regulate EZH2 methyltransferase activity [15, 30]

Cell division is a highly regulated process through which 2 genetically identical daughter cells are created from a single mother cell through the partition of various cytoplasmic and nuclear components [82]. At a high level, cell division begins with chromosome replication which is followed by an equal chromosomal division, such that each daughter cell inherits exactly one genetic copy of the parent chromosome. lncRNAs primarily influence cell division by modulating mitotic and cytokinetic protein levels, regulating specialized microtubule-associated protein (MAPs) expressions, and controlling kinetics of contractile (CN) ring formation as we discuss ahead (Fig. 4) (Table 2).

**Table 2:** Role of lncRNAs in cell division, cytoskeleton architecture, and cell cycle regulation

Long Non-Coding RNA	Target Proteins and Genes	Mechanism of Action	References
Linc00899	TPPP/p25	Interacts with chromatin regulators for fine-tuning TPPP levels for regulating oligodendrocyte differentiation	[104]
RI	PLK1	Regulates PLK1 stability by competing with it for binding to miR-210-3p to modulate cell division	[124]
CRYBR3	G-actin	Directly binds to the G-actin form and inhibits its polymerization causing M phase cell arrest that prevents CN ring formation	[84]
ARST	ALDOA	Negatively regulates the enzyme aldolase A (ALDOA) for exposing F-actin to Cofilin for its depolymerization for reducing glioma tumor	[106]
TUG1	α-actin and EZH2	Regulates cortex cytoskeleton by binding to both α-actin and EZH2 for regulating EZH2 methyltransferase activity	[15, 30]
miR503HG	Cyclin D1	Suppresses cyclin D1 expression for reducing carcinogenesis	[39]
Gadd7	TDP43, CDK6	Binds to TDP-43 protein for disturbing the communication its communication with CDK6 in response to DNA damage	[60]
KCNQ1OT1	p57	Suppresses p57 expression by binding to G9a and PRC2 chromatin-modifying complexes	[35]
NORAD	N/A	Dysfunction of cell cycle progression at multiple checkpoints occurs in cells depleted in the lncRNA NORAD	[76]
HCG11	IGF2BNP2	Recruits IGF2BP2 for enhancing p27 mRNA expression Acts as a ceRNA by restraining miR-942-5p expression for boosting levels of p27	[36]

### Influence of lncRNAs on mitotic spindle assembly

Mitotic spindles (MTs) are the most important macromolecular structures to ensure that chromosomal division occurs in a regulated way during mitosis. A major portion of mitotic spindle assemblies constitutes the microtubule bundles, which are distinct, preserved dimers of α- and β-tubulins [81]. MAPs control many characteristics displayed by microtubules via regulating MT polymerization-depolymerization kinetics and their PTMs [95]. For instance, the crucial transcription factor protein TPPP/p25 controls and stabilizes mitotic spindle assembly by boosting MT acetylation [95, 112, 113]. Increased and reduced cell proliferation are observed with mutations in the form of over- and underexpression of TPPP/p25 levels, respectively [96]. Furthermore, in mammalian cells, the downregulation of TPPP/p25 levels in progenitors depreciates oligodendrocyte differentiation [56, 71]. The lncRNA linc00899 is actively involved in fine-tuning TPPP/p25 expression for regulating mitosis by repressing TPPP levels. Linc00899's transcription site is spatially close to TPPP in the cell nucleus, which allows it to act swiftly and reduce TPPP levels by potentially interacting with chromatin regulators [104].

Mitosis is regulated by various cyclins/CDK complexes / CDK inhibitors. PLK1 gene is another Ser/Thr kinase whose activity is a critical regulator of various cell division events including but not limited to mitotic spindle assembly, cytokinesis, and chromosome division [98]. Abnormal or overexpressed PLK1 expressions are associated with cell division arrest, aberrant mitotic spindle designs, and DNA anaplasty. The lncRNA—RI modulates PLK1 expressions, to control mitosis and ensures a structured spindle organization. By protecting the PLK1 mRNA 3′UTR through sponging miRNA-210-3p, Lnc-RI controls the stability of the PLK1 mRNA expressions and by extension cell division [124].

### Role of lncRNAs in cytokinesis and cytoskeleton architecture

During cytokinesis, the mother cell is physically divided by a complex contractile network (CN) ring made up of actin filaments, non-muscle myosin II, and other essential components like α-actin [78]. Based on ATP binding and ATP hydrolysis, actin dynamically interchanges forms between the monomeric G-actin and polymeric filamentous F-actin forms, and this transition is crucial for cytokinesis [105]. The lncRNA LNC CRYBR3 directly binds to the G-actin form and inhibits its polymerization causing M phase cell arrest that prevents CN ring formation. The serum response factor (SRF) is a transcription factor having DNA binding domains on its surface, allowing it to form structural complexes to promote interactions with several other transcriptional regulators to control cytoskeletal actin [72]. MAL is a 17 kDa protein that specializes in the formation of condensed membrane domains and is frequently overexpressed in various carcinomas [92]. During the M phase or in the event of excessively high levels of LNC CRYBG3, LNC CRYBG3 binds to G-actin and MAL,preventing the accumulation of MAL in the nucleus that inactivates SRF and its downstream genes. As a result, the cells are unable to form a CN ring to carry out cell division and are stuck in the M phase. Clinically, the LNC CRYBR3 gene holds great promise, as its overexpression induces a programmed cell death causing the inhibition of invasive proliferation in lung cancer [84].

The lncRNA ARST downregulates the enzyme aldolase A (ALDOA) for establishing control over F-actin formation. ALDOA has a strong tendency to bind to F-actin and plays a very important role in its polymerization and stabilization. Intriguingly, both ALDOA and F-actin are often overexpressed in glioma tumor cells and enhance their metastatic potential. Cofilin is another actin-binding filament that regulates and enhances the depolymerization of actin fibers. Overexpressing ARST obstructs the binding of ALDOA to actin fibers; exposing the F-actin binding sites to cofilin, accelerating their depolymerization and hence the development of new actin fiber elements is significantly hindered [106].

RAC1, a member of the Rho GTPase family, functions as a cytoskeleton morphology regulator that is overexpressed in various carcinomas and plays an important role in all facets of cancer growth including proliferation, metastasis, and treatment resistance [57]. Notably, the lncRNA MEG3 antagonizes RAC1 expression in thyroid carcinoma by interacting with its 3’-UTR mRNA to function as a RAC1 tumor inhibitor [120].

Curiously, RNA pull-down assays performed on rat vascular smooth muscle cells (VSMCs), reveal that the lncRNA TUG1 physically binds to both α-actin and EZH2 for regulating EZH2 methyltransferase activity. This action by TUG1 on α-actin is a necessary prerequisite for cortex cytoskeleton formation and silencing TUG1 disrupts its interaction with α-actin and EZH2 leading to increased depolymerization of F-actin in VMSCs [15, 30]. Indeed these studies have highlighted novel biophysical pathways by which lncRNAs positively influence cytoskeleton architecture.

### lncRNAs regulate multiple checkpoints in the cell cycle

**Fig. 5:**
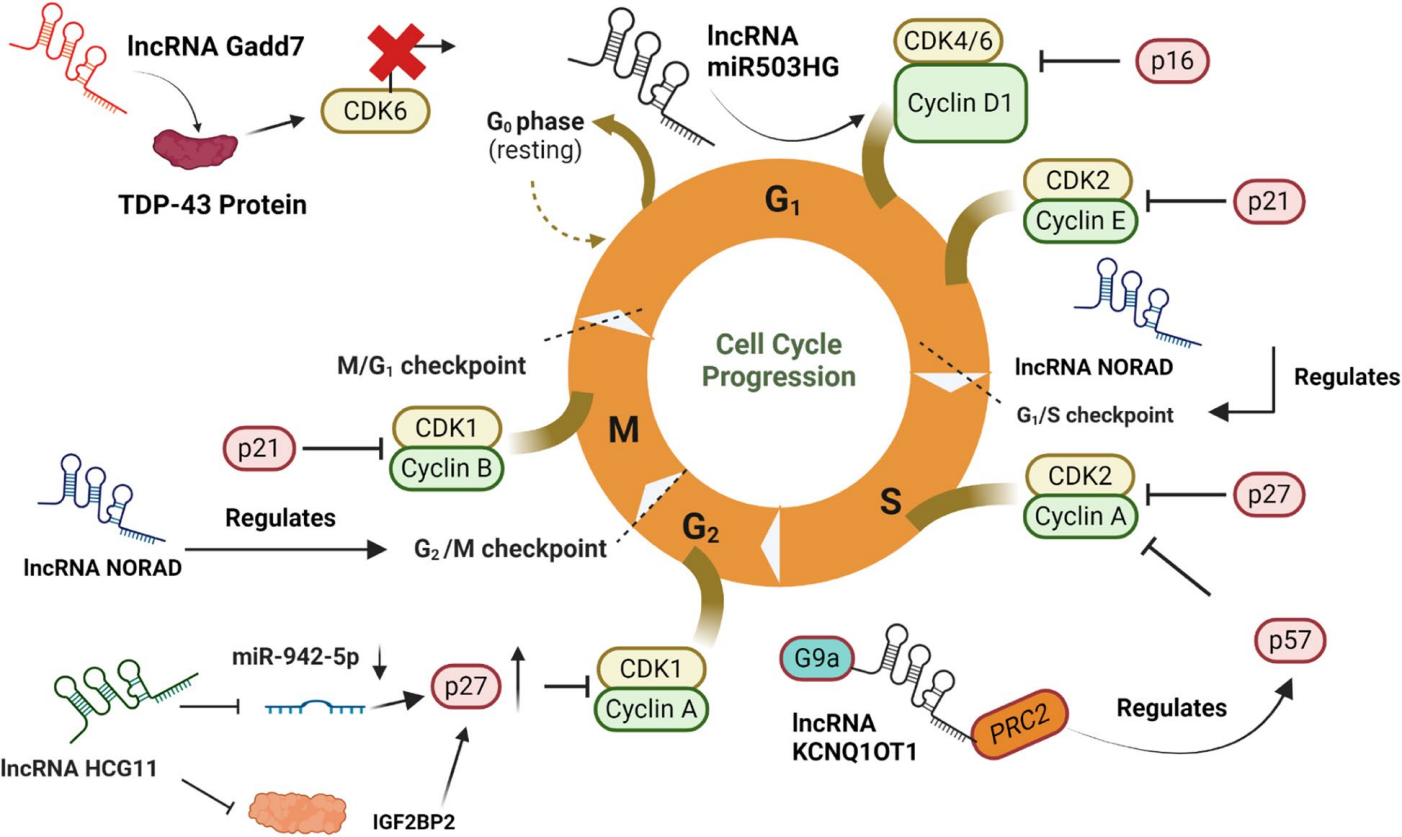
lncRNAs Regulate Cyclin and Cyclin Inhibitor Expressions in Cell Cycle. **A** | lncRNA miR503HG prevents cancer progression by targeting and suppressing cyclin D1 expression [39] **B** | lncRNA gadd7 dysregulates the interaction between TDP-43 and the cyclin CDK6 by interacting with the TDP-43 protein leading to mRNA decay of CDK6 [60]. **C** | lncRNA KCNQ1OT1 cis-silences p57 expression by binding to PRC2 and G9a chromatin regulators [35] **D** | lncRNA HCG11 functions as an osteosarcoma suppressor by repressing miR-942-5p expression to boost p27 levels. Secondly, it recruits IGF2BP2 which consequently enhances p27 mRNA expression [36]

The cell cycle is a complex, dynamic system that is necessary for the development and proliferation of cells. In different stages of the cell cycle, CDK1, CDK2, and CDK4/6 are transiently activated by binding to their selected cyclins, including cyclins A, B, D, and E. Thereafter, the complexes phosphorylate their target proteins to drive cell cycle advancement [53]. The mitogens that subdue the E2F target genes using the CDK4/6-cyclin D in the early G1 phase and consequent upregulation of CDK2-cyclin E driven by E2F, coupled with the CDK1-cyclin A/B expression in the G1/S and G2/M stages, respectively, constitute the backbone that drives cell cycle progression [109] (Fig. 5).

### Modulating cyclin expressions in the cell cycle

lncRNAs directly and indirectly control various CDK/cyclins expressions. For example, lncRNA miR503HG suppresses cyclin D1; known to boost expressions of the G1/S cell cycle which is found to be a leading cause in cancer progression, and lowering levels of miR503HG is attributable to poor prognosis and survival in cancer patients [39]. In response to DNA damage, genomic integrity must be maintained to ensure the proper functioning of the cell cycle. A novel lncRNA gadd7 overexpressed in response to UV radiation modulates the G1/S checkpoint by interacting with the TDP-43 protein to dysregulate the interaction between TDP-43 and the cyclin CDK6 leading to mRNA decay of CDK6 [60].

The lncRNA HCG11 functions as an osteosarcoma suppressor, by upregulating p27 Kip1 (p27) expression. p27 is a universal CDK inhibitor that functions as an important tumor suppressor gene and is often underexpressed in tumor development stages [63]. Recently, [18] demonstrated that in osteosarcoma (OS), cytoplasmic p27 protein can be synthetically overexpressed by chemotherapeutic agents to boost apoptosis of cancer cells. Besides chemotherapeutic intervention, another study by [36], provides novel intriguing insights into lncRNA-mediated p27 enhancement. The lncRNA HCG11 is significantly underexpressed in OS and this is responsible for poor prognosis and survival in patients. Studies conducted both in vivo and in vitro prove that HCG11 enhances p27 expression by a dual mechanism. Firstly, by directly binding to and restraining miR-942-5p expression and secondly, by recruiting IGF2BP2 protein which enhances the stability of p27 mRNA.

### Controlling cyclin inhibitor expressions

lncRNAs can influence cyclin inhibitor expressions to regulate the cell cycle. An interesting case pertains to the lncRNA KCNQ1OT1. p57 is a cyclin-dependent kinase inhibitor and is a critical regulator of the mammalian cell cycle that negatively regulates CDK activity and depreciates the formation of all forms of cyclin CDK complexes. In hematopoiesis, p57 functions as the principal controller of tissue differentiation and plays an important role in the timing of the cell cycle exit of myeloid progenitors [6, 91]. The lncRNA KCNQ1OT1 controls p57 gene expression by actively suppressing its production levels using cis-silencing by binding to PRC2 and G9a chromatin regulators [35]. Despite having an exclusive paternal expression in mammalian tissues as an antisense RNA of the KCNQ1 domain [53], KCNQ1OT1 is programmed to control both maternal and paternal p57 alleles by interacting with the chromatin on the intragenic regions of p57 and deletion of KCNQ1OT1 causes overexpression of maternal p57 which can result in severe disabilities like the Beckwith-Wiedemann syndrome [2, 38].

Mutations in lncRNA expressions serve as one of the reasons for cell cycle dysfunction and the onset of tumorigenesis. A phenomenal study by [76] highlighted the dysfunction of cell cycle progression at multiple checkpoints in cells depleted in the lncRNA NORAD. Fluorescence-activated cell sorting (FACS) revealed a stark downregulation in the S phase while a simultaneous increase in G1 phase levels was observed in cells with silenced NORAD expression. The authors further concluded that chromosomal aberrations observed in NORAD-depleted cells can induce cell cycle arrest in the G1 phase. Furthermore, mutations in the form of overexpressed G2/M phase are also induced in cells with NORAD lacking RBMX protein binding sites, which conclusively proves that NORAD can only function effectively when RBMX binding sites are present in the structure. This further reaffirms the structure–function relationship of lncRNAs that we previously discussed in detail in Sect. 2.2 of our review.

### lncRNAs as crucial regulators of hematopoietic differentiation

**Fig. 6:**
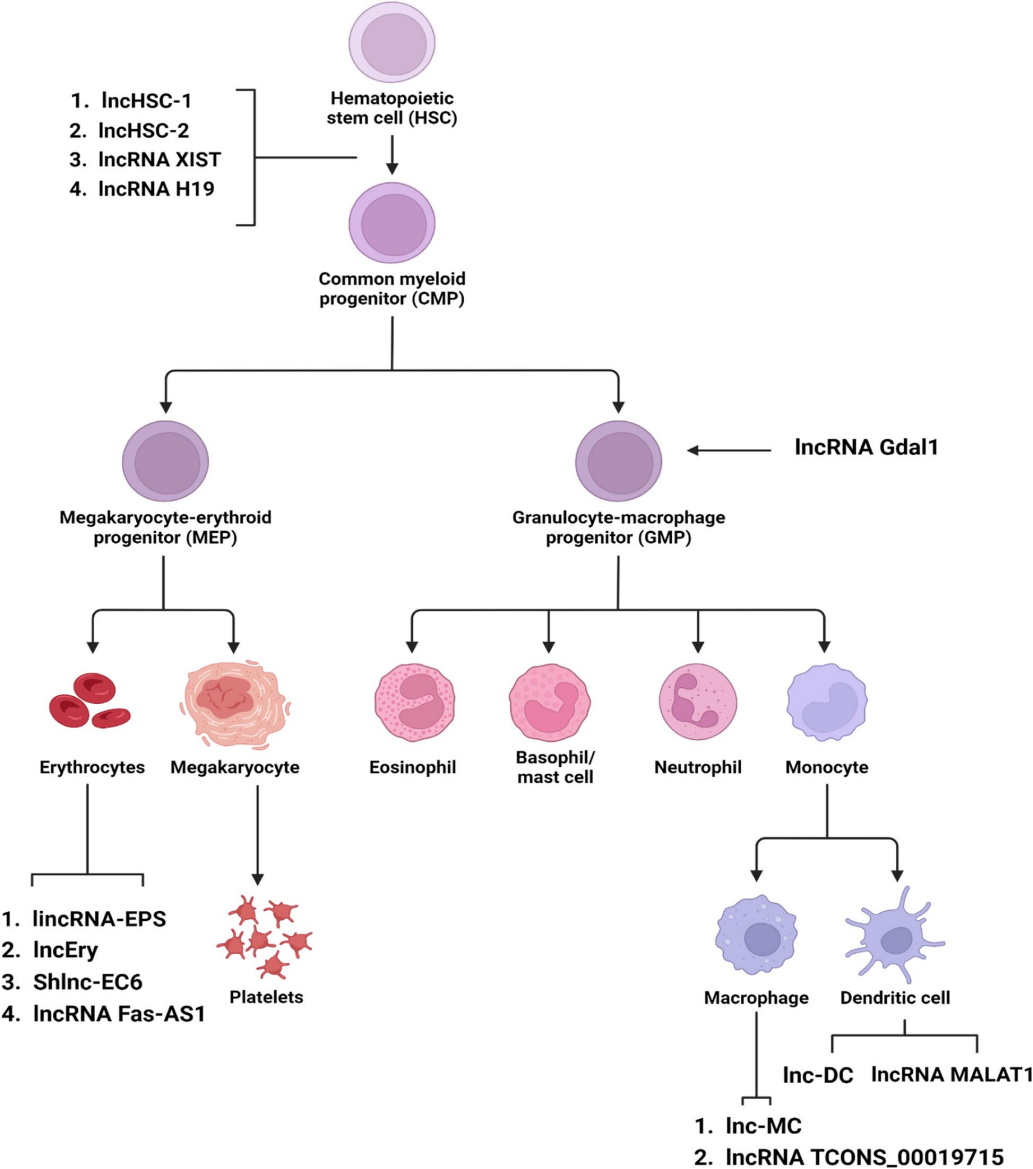
Hematopoiesis Control By lncRNAs. The above figure summarizes the role of the most important polyadenylated and non-polyadenylated lncRNAs in hematopoietic cell differentiation and maturation

Hematopoiesis is the highly regulated process of development of various blood cell lineages from hematopoietic stem cells (HSCs) that undergo multiple, intermediate stages of maturation [22]. lncRNAs are involved in almost every step of hematopoiesis right from HSC differentiation to myeloid and lymphoid maturation respectively. RNA-seq studies conducted on hematopoietic stem cells (HSCs), differentiated B-cells, and granulocytes revealed that two nuclear lncRNAs—lncHSC-1 and lncHSC-2 are involved in regulating cell differentiation. In vivo experiments revealed that these two lncRNAs are primarily involved in lineage differentiation. lncHSC-1 influences myeloid differentiation while lncHSC-2 controls the regenerative capacity of HSCs and T-cell differentiation [64] (Fig. 6) (Table 3).

**Table 3:** Role of lncRNAs in hematopoiesis

Long Non-Coding RNA	Target Proteins and Genes	Mechanism of Action	References
LncHSC-1	N/A	Regulates myeloid differentiation	[64]
LncHSC-2	N/A	Influences regenerative capacity of HSC and T-cell differentiation	
H19	Igf2, Runx1, Spi1	Suppresses the maternal allele of Igf2 via Igf2-Igf1r pathway for long-term maintenance of HSCs Promotes embryonic development by demethylating transcription factors like Runx1 and Spi1	[117, 139]
XIST	N/A	Involved in X-chromosome silencing	[133]
lincRNA-EPS	PyCard	Suppresses Pycard expression for ensuring erythrocyte differentiation by preventing cell apoptosis	[47]
lncEry	KLF1 and Globin Genes	Facilitates binding of H3K4me3 to the CRRs of KLF1 and to the CRRs and LCRs of globin genes for erythrocyte differentiation	[130]
shlnc-EC6	Rac1	Interacts with 3’-UTR of Rac1 mRNA to inhibit PI5K expression for regulating erythropoiesis	[119]
Fas-AS1	N/A	Protects erythroblasts from Fas modulated cell death signals	[118]
Gdal1	N/A	Involved in granulocytic differentiation	[55]
lnc-MC	miR-199a-5p	Targets miR-199a-5p for regulating the differentiation of monocytes to macrophages	[14]
TCONS_00019715	N/A	Regulates macrophage polarization to a pro-inflammatory (IFN-γ + LPS) phenotype	[43]
lnc-DC	STAT3	Binds with STAT3 to prevent its interaction with SHP1 for promoting dendritic cell differentiation	[122]
MALAT1	miRNA-155	Sequesters miRNA-155 to promote DC-SIGN expression to regulate immune tolerance in dendritic cells	[126]

### Control of genome imprinting

IGF2 is a biallelic (both maternal and paternal expression) gene that is overexpressed in long-term HSCs and is an important element for their maintenance, differentiation, and maturation [110]. The lncRNA H19 is well known for genome imprinting to regulate hematopoiesis. The H19-differentially methylated region (H19-DMR), which is upstream of the H19 TSS uses both the alleles of IGF2 to control the expression of H19 which further maintains HSC quiescence by ensuring that the IGF2-IGF1R pathway remains dormant. When the maternal allele of IGF2 is preferentially suppressed via the IGF2-IGF1R pathway, adult hematopoietic cell dormancy is reduced consequently hindering HSC function. Mechanistically, knockdown of the maternal H19-DMR allele leads to heightened IGF2 and IGF1R translation, which causes phosphorylated FOXO3 to move from the nucleus to the cytoplasm and causes FOXO3-regulated cell cycle arrest, ultimately resulting in the collapse of HSCs [117].

Another interesting study revealed the climactic role played by H19 in embryonic HSC evolution. It was found that H19 regulated embryonic development by promoting pre-HSC and HSC states through demethylating the promoters of various hematopoiesis-associated master transcription factors like RUNX1 and SPI1. Both RUNX1 and SPI1 are indispensable transcription factors that play crucial roles in the process of endothelial-to-hematopoietic transition (EHT) as well as both the development and maintenance of HSCs [48]. This mechanism is proposed by the observation that pre-HSCs with dormant H19 advertised a significant concordant downregulation of the aforementioned transcription factors. Mechanistically, the downregulation can be moderately attributed to the overexpression of the S-adenosylhomocysteine hydrolase enzyme [137, 139].

### Erythropoiesis associated lncRNAs

Erythropoiesis is the process when HSCs mature and proliferate into red blood cells (RBCs) [9]. Indeed, studies have screened and identified various lncRNAs specifically involved in regulating this process like the lincRNA-EPS which prevents cellular apoptosis moderately by suppressing Pycard expressions to ensure erythroid cell differentiation [47]. The erythropoiesis-specific transcription factor KLF1 functions as a molecular scaffold on which processes such as SUMOylation, ubiquitination, acetylation, and phosphorylation allow its interaction with STAT family-associated proteins and chromatin-modifying complexes like SWI/SNF to consequently facilitate erythropoiesis [10]. A novel lncRNA lncEry localized in the nucleus has recently been discovered to be of great importance during the early and terminal stages of erythropoiesis by controlling KLF1 expression. LncEry functions as a facilitator of H3K4me3 binding to the cis-regulating regions (CRRs) of the transcription factor KLF1 and the CRRs and locus control regions (LCRs) of globin genes by directly binding to Wdr82 for modulating erythropoiesis [130].

Another lncRNA shlnc-EC6 regulates erythropoiesis by a novel RAC1/PIP5K signaling pathway. An important prerequisite to ensure successful erythrocyte differentiation is enucleation. Enucleation is a complex process in the final stages of RBC production that leads to the formation of reticulocytes (immature RBCs) from orthochromatic erythroblasts [69]. Shlnc-EC6 is overexpressed in reticulocytes and silencing its expression hinders the enucleation process. From a mechanistic standpoint, shlnc-EC6 post-transcriptionally inhibits RAC1 expression (an enucleation inhibitor) by attacking the 3’-UTR site of RAC1 mRNA and consequently constraining PIP5K expression. Therefore shlnc-EC6/RAC1/PIP5K signaling axis can potentially serve as an important therapeutic target from a clinical standpoint for treating malignant hematopoietic diseases [119]. Finally, the overexpression of another erythroid-specific lncRNA Fas-AS1, induced due to activities by KLF1 protects erythroblasts obtained from CD34^+^ HSC/HPCs of healthy donors from Fas-regulated cellular apoptosis signals [118].

### Myelopoiesis associated lncRNAs

lncRNAs influence myelopoiesis including Gdal1 involved in granulocytic differentiation [55]. lnc-MC is involved in regulating the differentiation of monocytes into macrophages to modulate immune response [14]. Apart from just regulating the macrophage differentiation process, lncRNAs also regulate macrophage polarization to a pro-inflammatory (IFN-γ + LPS) phenotype,an expression of critical control during an inflammatory response, as seen in the case of lncRNA TCONS_00019715 [43]. The production and immune tolerance of dendritic cells are essential for preparing the immune system against attacks from external microbes and the extensively studied lncRNAs known to govern this process are lnc-DC [122] and MALAT1 [126]. What remains unknown as a potential topic for future research is the role of lncRNAs in the trans-differentiation of cells from one functional form into another. Recent attempts have been made to explore how lncRNAs affect trans-differentiation [88]. High throughput RNA seq conducted by [87] revealed that 114 lncRNAs were annotated as crucial regulators of adipogenic muscle cell transdifferentiation.

### Concluding remarks

The recent advances in transcriptomic and proteomic technologies have revolutionized our understanding of the human genome. This is well complemented by a greater understanding of the structure and functions of lncRNAs and scientists have been cognizant of their appreciation for the multidimensional role of these molecules in regulating various physiological, pathophysiological, and pathological processes. In light of this, we must also not forget that we are still in the infancy stage of unraveling the true potential of lncRNAs as master genetic regulators. A lot of scientific investigation is still warranted owing to the low conservation and lack of understanding of the structure–function relationship of lncRNAs. It can be stated without a shadow of a doubt that investigating more about how lncRNAs affect complex processes in the human body and their role in various diseases and malignancies is of great importance to current genetic research.

## Data Availability

Data sharing does not apply to this article as no new data were created or analyzed in this study.
